# Total Gestational Weight Gain Is Explained by Leptin and Body Fat, Regardless of Pre-Pregnancy Body Mass Index and Other Adipokines, in Mexican Adolescents

**DOI:** 10.3390/nu16132147

**Published:** 2024-07-05

**Authors:** Gabriela Chico-Barba, Reyna Sámano, Hugo Martínez-Rojano, Rosa María Morales-Hernández, Edgar Barrientos-Galeana, Andrea Luna-Hidalgo, Martha Kaufer-Horwitz, Gregorio T. Obrador, Antonio Rafael Villa-Romero

**Affiliations:** 1 Programa de Maestría y Doctorado en Ciencias Médicas, Odontológicas y de la Salud, Facultad de Medicina, Universidad Nacional Autónoma de México, Mexico City 04510, Mexico; gabyc3@gmail.com; 2 Coordinación de Nutrición y Bioprogramación, Instituto Nacional de Perinatología, Mexico City 11000, Mexico; 3 Sección de Posgrado e Investigación, Escuela Superior de Medicina, Instituto Politécnico Nacional, Mexico City 11340, Mexico; hmartinez_59@yahoo.com.mx; 4 Dirección de Nutrición, Instituto Nacional de Ciencias Médicas y Nutrición Salvador Zubirán, Mexico City 14080, Mexico; 5 School of Medicine, Universidad Panamericana, Mexico City 03920, Mexico; 6 División de Investigación, Facultad de Medicina, Universidad Nacional Autónoma de México, Mexico City 04510, Mexico

**Keywords:** leptin, adiponectin, irisin, C-reactive protein, teenage pregnancy, inflammation, gestational weight gain

## Abstract

Pre-pregnancy body mass index (pBMI) is a predictor of gestational weight gain (GWG). However, other factors, such as adipokines and inflammation markers, may also be associated with GWG. The aim of the study was to determine the association of leptin, adiponectin, irisin, and C-reactive protein, with GWG in adolescents. A longitudinal study was conducted from 2018 to 2023 in adolescents with a clinically healthy pregnancy. The assessments included sociodemographic and clinical data, pBMI, percent of body fat, serum concentrations of leptin, adiponectin, irisin, and high-sensitivity C-reactive protein (hsCRP), and total GWG adequacy. Cox regression models were performed, the outcome variables were inadequate and excessive GWG. In 198 participants, being overweight/obesity was marginally associated with a protective effect against inadequate GWG (HR = 0.44, 95%CI = 0.18–1.06), regardless of maternal characteristics and adipokines. Leptin (HR = 1.014, 95%CI = 1.008–1.021), and body fat percent (HR = 1.11, 95%CI = 1.05–1.17) were associated with a higher risk of excessive GWG, independent of other maternal variables such as pBMI, while adiponectin was associated with a lower risk. These findings suggest that, in Mexican adolescents, adipose tissue and its adipokines during pregnancy may play a more significant role in the final GWG than body weight.

## 1. Introduction

Adolescent pregnancy is a public health problem in low- and middle-income countries [1]. Mexico ranks among the highest in Latin America [2], with approximately 15.3% of total births attributed to women under 20 years old [3]. Teenage mothers face increased risk of adverse perinatal outcomes, including preeclampsia, infections, postpartum hemorrhage, and maternal mortality [4,5,6]. Additionally, infants born to adolescent mothers are more likely to experience low birth weight, be small for gestational age, and experience intrauterine growth restriction [4,5,6].

Gestational weight gain (GWG) may play a role in the aforementioned perinatal outcomes [7,8,9]. GWG is predominantly explained by the increase in fat mass [10] and is regulated by diet, exercise, and maternal characteristics such as age, pre-pregnancy body mass index (BMI), and metabolic factors [10]. Among these metabolic factors, adipokines such as leptin [11,12,13], adiponectin [12,14], and the adipomyokine irisin [15], may be associated with both inadequate and excessive GWG. Leptin and adiponectin are the most studied adipokines in pregnancy outcomes and GWG, but there are others, such as chemerin, resistin, and visfatin with scarce evidence [16].

Leptin, secreted by white adipose tissue, regulates metabolic and endocrine functions [17]. During pregnancy, the placenta also secretes leptin, leading to hyperleptinemia and leptin resistance, thus contributing to changes in energy balance [18,19]. Evidence suggests that higher leptin serum concentrations are associated with excessive weight gain in adolescents [20,21]. However, studies describing the mechanisms by which leptin may regulate GWG are limited [22]. For instance, one proposal is that central regulation of appetite in the hypothalamus is altered in leptin resistance, favoring increased food intake and more weight gain [23].

In contrast, adiponectin, another hormone derived from white adipose tissue with actions opposite to leptin, decreases during pregnancy and exhibits anti-inflammatory properties on microglia cells [14,22,24,25].

Irisin, a novel hormone primarily secreted by skeletal muscle during exercise but also by fat tissue, is considered an adipomyokine [26]. Its levels increase during pregnancy and it regulates maternal and fetal glucose levels, with most studies focusing on its relation to gestational diabetes [27,28,29]. Although there is no evidence for the association of irisin and GWG, its role in the conversion of white to brown adipose tissue, stimulated by exercise [26], makes irisin a promising adipomyokine to be studied in the context of gestational weight outcomes.

Limited evidence exists regarding the role or association of adiponectin and irisin in GWG in both adults and adolescents [12,30].

Furthermore, leptin and irisin act as pro-inflammatory hormones [17,31], while adiponectin exhibits anti-inflammatory properties [24]. Pregnancy itself induces inflammation [32], as does obesity in non-pregnant individuals due to the secretion of pro-inflammatory proteins by adipose tissue [31]. High-sensitivity C-reactive protein (hsCRP), the principal and most studied inflammatory marker, increases in both scenarios [33,34], with reported increments of 3% in hsCRP for every 1 kg increase in gestational weight [35].

Moreover, the prevalence of overweight and obesity among Mexican female adolescents is approximately 41% [36], predisposing them to begin pregnancy with higher body fat levels. Pre-pregnancy overweight and obesity are associated with excessive weight gain during pregnancy [37,38].

Adipokines, such as leptin, adiponectin and irisin, are mediators of the inflammatory response and metabolic regulation. Although the mechanisms are not fully described, leptin and other adipokines could be prognostic factors for metabolic diseases and for events such as insufficient or excessive weight gain during pregnancy. Studies on the association between adipokines and GWG focused on adult women in high-income countries or in nutritional and health contexts different from those of Mexican adolescents [11,12,13,20,25,39]. It is necessary to identify these biomarkers in adolescents since their levels of adipokines and inflammation markers could vary due to the stage of fat mass accretion they are in, in addition to the changes caused by pregnancy. Increased fat tissue before or during pregnancy predisposes women to retain weight, and it predisposes offspring to metabolic diseases, as the maternal pregnancy metabolic and inflammatory environment plays a crucial role in the early origins of disease [40,41,42,43]. Identifying metabolic and inflammatory factors related to GWG is essential for understanding the mechanisms predisposing young girls to gain more or less weight than recommended during pregnancy. Therefore, this study aimed to determine the association of leptin, adiponectin, irisin, and hsCRP with total gestational weight gain in adolescents, evaluating their potential as prognostic factors.

## 2. Materials and Methods

### 2.1. Study Design and Sample Collection

We conducted a follow-up study at the Instituto Nacional de Perinatología (INPer) in Mexico City from 2018 to 2023. Participants were selected through convenient sampling based on consecutive cases meeting the following inclusion criteria: pregnant adolescents aged 10–19 years (as defined by the World Health Organization [44]), receiving medical antenatal care at INPer, primigravida, and with a healthy singleton pregnancy. All participants were recruited during their outpatient consultation at INPer. Exclusion criteria were having pre-pregnancy chronic or inflammatory diseases such as type I or II diabetes, cardiac, kidney, autoimmune, or psychiatric diseases, as well as the consumption of alcohol, tobacco, or drugs during pregnancy. The sample size was calculated with a 95% confidence level, based on a correlation coefficient of 0.26 between leptin and gestational weight gain in adolescents [20], as leptin is the most studied adipokine. The calculated sample size was 114 participants. However, accounting for an expected 15% loss rate, a total of 131 participants was required.

### 2.2. Sociodemographic and Clinical Variables

During the initial assessment, with one legal guardian present, information on age, occupation, marital status, education, and socioeconomic status was collected through a questionnaire. Menarche age and gestational age, determined by last menstrual period to determine the trimester of initiation of prenatal care, were obtained from medical records. Physical activity was asked as a direct question, with a yes or no response. Dietary intake was assessed using a single 24-h recall; total energy intake (kcal/d) was determined in the Nutrickal^®^ software https://www.nutrikcal.mx/NutriKcalVO.html (Mexico City, Mexico). The sociodemographic, anthropometric, clinical, and dietetic measurements were performed by six trained nutritionist.

### 2.3. Anthropometric Evaluation

In the first visit, weight before pregnancy was self-reported, which is an accurate measurement of real pre-pregnancy weight [45,46]. In addition, height was measured with a manual stadiometer (SECA 222, Hamburg, Germany 0.1 cm accuracy).

Pre-pregnancy body mass index (pBMI) was calculated using pre-pregnancy weight and height; then, pBMI classification was obtained with AnthroPlus^®^ https://www.who.int/tools/growth-reference-data-for-5to19-years/application-tools (World Health Organization, Geneva, Switzerland) according to z-scores: underweight ≤ −2, normal weight −2 to +1, overweight 1 to 1.99, and obesity >+2 [47].

Body fat percent was obtained using an InBody 770^®^ Body Composition Analyzer (InBody Co., Ltd, Seoul, Korea). This assessment was performed during morning hours (from 8:00 to 10:00 h) in fasting conditions, with an empty bladder and light clothes.

Maternal weight was measured using a digital scale (TANITA, Tokyo, Japan, model BWB-800; 0.010 kg accuracy). Final weight was assessed one or two weeks before delivery. We calculated total GWG by the difference between the last measured weight and the pre-pregnancy weight.

Then, to assess the GWG as a continuous variable, we calculated the percentage adequacy of GWG by dividing the total GWG by the expected GWG and multiplying the result by 100 [48]. The expected GWG was obtained with the following formula: expected weight gain = recommended weight gain for the first trimester + ((gestational age final − 13.86 weeks) × (recommended weight gain rate in second and third trimesters)). According to IOM recommendations, the recommended GWG rates were based on pBMI as follows: low and normal weight 2 kg, overweight 1 kg, and obesity 0.5 kg for the first trimester, and low weight 0.51 kg, normal weight 0.42 kg, overweight 0.28 kg, and obesity 0.22 kg/week for second and third trimesters [10].

To assess the GWG as a categorical variable, the percentage adequacy of GWG was categorized into inadequate (<90%), adequate (90 to <125%), and excessive (≥125%) [49].

### 2.4. Biochemical Determinations

A nurse collected blood sample between 7 and 8 a.m., after fasting 8 to 10 h. Then, serum was obtained after centrifugation for 10 min at 3500 rpm; samples were frozen at −70 °C until processing. Leptin, adiponectin and irisin were determined by ELISA technique.

For leptin and adiponectin, we used an absorbance microplate reader (ELISA Bio-Rad, model 680 Bench-mark Plus, Bio-Rad, Hercules, CA, USA) and ELISA Human Immunoassay kit (Quantikine^®^ ELISA Human Immunoassay, R&D Systems Inc., Minneapolis, MN, USA), respectively, following manufacturer’s instructions.

Irisin was determined using the Human irisin ELISA kit (My BioSource.com, MBS 706887, San Diego, CA, USA).

High-sensitivity C-reactive protein (hsCRP) was measured by colorimetric method (Respons 910, DiaSys Diagnostic Systems, Holzheim, Germany) with a detection range of 0.1 mg/L.

The biochemical determinations were performed by three clinical chemist.

### 2.5. Statistical Analyses

Frequencies, measures of central tendency and dispersion were obtained to describe the sample characteristics. Kruskal–Wallis and Mann–Whitney U tests were used to compare serum adipokines concentrations according to pBMI and GWG category. Additionally, Spearman’s Rho was calculated to correlate the biochemical variables.

To determine the association of the serum concentrations of the adipokines with GWG, we performed Cox regression models, where time was determined in days (gestational age minus weeks of gestation at the moment of blood sample); the outcome variables were inadequate and excessive GWG. Both models were adjusted by pBMI, maternal age, physical activity, and energy intake. Hazard ratios with 95%CI were obtained.

A sensitivity analysis was performed in participants that started the study between 18 and 30 weeks of gestation, as the metabolic and inflammatory status are similar during these weeks. In this way, we excluded the effect of gestational age in the adipokines status.

All the analyses we performed using IBM SPSS Statistics for Windows, version 23.0 (IBM Corp., Armonk, NY, USA).

### 2.6. Ethical Aspects

The study was approved by the Institutional Ethics, Biosafety, and Research Committees (INPer registration number 2017-2-101). Written informed assent was obtained from the adolescents and written informed consent was obtained from their legal guardians. Confidentiality was guaranteed by assigning an ID number for each participant during data collection and analysis. All participants were informed that their medical care would not be affected by their participation in the study.

## 3. Results

**Figure 1:**
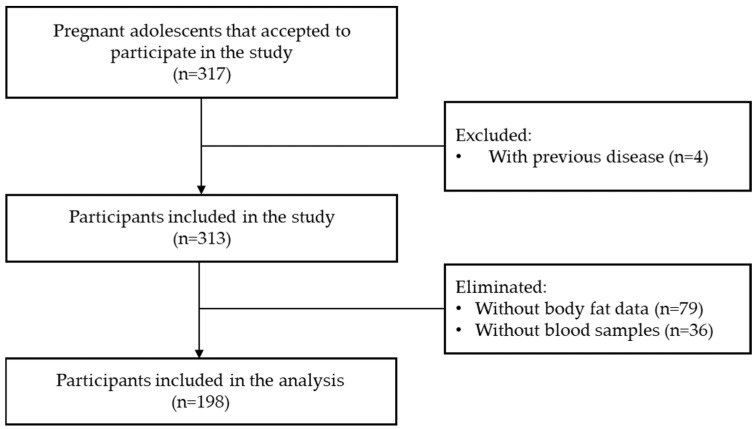
Flowchart of the participants’ selection.

A total of 198 adolescents were included in the analysis. Figure 1 shows the flow chart of participant selection. Table 1 shows the participants characteristics at the first visit. Mean age was 15.9 ± 1.4 years, most of them were single, homemakers and had a pBMI of normal weight.

**Figure 2:**
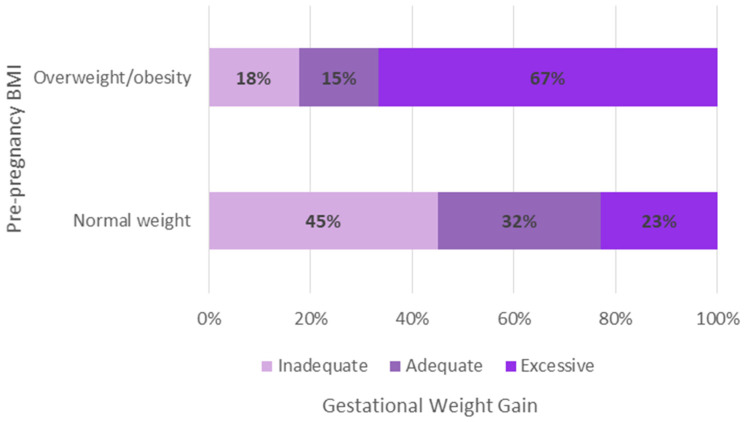
Total gestational weight gain according to pre-pregnancy body mass index.

Figure 2 shows the total GWG by pBMI. Inadequate GWG was most common in participants with pre-pregnancy normal weight, while excessive GWG was more frequent in those living with pre-pregnancy overweight/obesity.

### Leptin, Adiponectin, Irisin, hsCRP, and Total Gestational Weight Gain

The median concentrations of leptin, adiponectin, irisin, and hsCPR were 35.3 ng/mL, 8627.9 ng/mL, 669.1 ng/mL, and 3.4 mg/L, respectively (Table 1).

Among adolescents with normal pBMI, differences in median concentrations were observed for leptin and adiponectin, between GWG categories (Kruskal–Wallis test, *p* = 0.023 and *p* = 0.001). Irisin showed almost statistical significance (*p* = 0.076).

For participants with overweight/obesity pBMI, only leptin median concentrations differed significantly between GWG categories (Kruskal–Wallis test, *p* = 0.038).

The correlations among the anthropometric and biochemical variables can be seen in the Supplementary file (Table S1).

**Figure 3:**
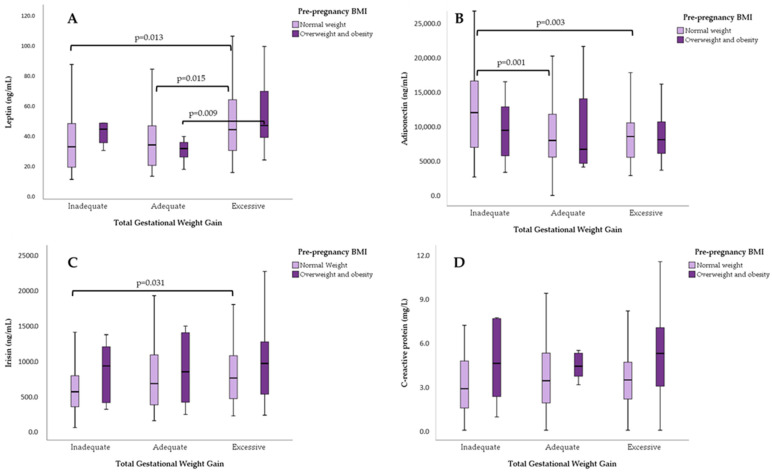
Serum concentrations of (**A**) leptin, (**B**) adiponectin, (**C**) irisin, and (**D**) hs-CRP, according to pre-pregnancy BMI and total gestational weight gain. U-Mann–Whitney test for pair comparisons.

Figure 3 shows the serum concentrations of each adipokine based on pBMI and GWG categories. In individuals with a normal pBMI, leptin concentrations differed significantly between the inadequate and excessive GWG groups, as well as between the adequate and excessive GWG groups. Among those living with pre-pregnancy overweight or obesity, significant differences in median leptin concentrations were observed only between the adequate and excessive GWG groups. For adiponectin, differences were found only among the GWG categories in women with a normal pBMI. Lastly, in adolescents with a normal pBMI, irisin levels differed between the inadequate an excessive GWG groups. No differences in hsCRP levels were found between the GWG categories.

According to the Cox regression models, pre-pregnancy overweight/obesity showed a nearly statistically significant association as a protective factor (HR = 0.44, 95%CI = 0.18–1.06) for inadequate GWG, regardless of maternal characteristics and the other adipokine concentrations, (see Table 2). On the other hand, leptin (HR = 1.014, 95%CI = 1.008–1.021), adiponectin (HR = 0.99994, 95%CI = 0.99988–0.99999), and body fat (HR = 1.11, 95%CI = 1.05–1.17) were associated with higher risk of having excessive GWG, as shown in Table 3.

The sensitivity analysis was performed in 126 participants who initiated the study from 18 to 30 weeks of gestation. The calculated hazard ratios showed similar associations for both outcomes, inadequate and excessive total GWG.

## 4. Discussion

In this observational study conducted on adolescents attending INPer in Mexico City, serum leptin concentrations were associated with a higher risk of excessive GWG along with body fat, while adiponectin was associated with a lower risk. Increases in leptin concentrations were particularly observed in adolescents living with pre-pregnancy overweight or obesity. None of the adipokines were associated with inadequate GWG, except for pre-pregnancy overweight and obesity, which were associated with a lower risk. Irisin and hsCRP showed no association with either inadequate or excessive GWG.

We found that 38.9% of the participants had inadequate GWG and 32.8% had excessive GWG. These results are very similar to the various frequencies of gestational weight gain in pregnant adolescents reported in different parts of the world, with fluctuations between 56% and 84% of adolescents typically experiencing inadequate gestational weight gain [50,51,52]. Therefore, inadequate gestational weight gain in pregnant adolescents is a health issue, as adolescents are a vulnerable group whose longitudinal growth is compromised [53,54,55] and they generally consume diets that are either excessive or deficient in quantity and/or quality [56], in addition to being exposed to adverse psychosocial risk factors that can influence gestational weight gain [57].

When GWG categories are divided by pBMI, we observed that excessive GWG was significantly higher in those living with overweight and obesity before pregnancy (67%), compared to those with normal weight (23%). These results are in accordance with previous findings from our study group [58], where we demonstrated that, in a group of adolescent mothers compared to adult mothers, adolescents had a higher gestational weight gain in kilograms compared to adults. These observations align with the United States Institute of Medicine’s assumption that adolescents, especially the younger ones, are more likely to be categorized in a “lighter group” and therefore advised to gain more weight [10,59]. Regarding the evidence of the effects of pre-pregnancy body mass index and gestational weight gain on maternal and neonatal outcomes, it was reported that being underweight before pregnancy increases the risk of preterm birth and delivering a small-for-gestational-age newborn. On the other hand, overweight and obesity are high-risk factors for gestational diabetes, hypertensive syndrome, and fetal growth disorders. In terms of weight gain, women with insufficient gestational weight gain may experience anemia. Conversely, those with excessive weight gain have an increased risk of cesarean delivery, preeclampsia, gestational diabetes, blood transfusions, postpartum weight retention, and long-term obesity [60].

Since pre-pregnancy BMI determine GWG, we stratified serum concentrations of adipokines by these variables. We found that leptin concentrations were different across GWG categories among adolescents with normal weight pBMI, but among adolescents living with overweight/obesity before pregnancy, the only difference was found when comparing adequate vs. excessive GWG. Similar results were reported in several studies in adults [11,61,62,63], but few studies were performed in adolescents. For instance, Baratto, et al. [25], reported higher serum leptin concentration in Brazilian adolescents who started their pregnancy overweight or obese compared to adolescents who began their pregnancy with a normal body mass index. Therefore, we believe that leptin secreted by adipose tissue and the placenta is involved in the regulation of food intake, energy homeostasis, insulin secretion, and nutrient transport to the fetus, correlating with pre-pregnancy body mass index and adiposity [64]. In our study, adolescents with excessive gestational weight gain showed higher serum leptin concentrations, which is probably related to an abnormal accumulation of body fat, mainly visceral adipose tissue, leading to adipocyte dysfunction [65] and an alteration in adipokine profiles, where adiponectin decreases and leptin concentration increases [66]. Consequently, several studies reported that high leptin concentrations in the second and third trimesters of pregnancy correlate with excessive gestational weight gain [67].

Regarding the association of leptin with higher risk of excessive GWG, studies found a stronger association of leptin in the second trimester with excessive GWG in women living with overweight or obesity [11,13]. Our results are consistent with those reported by Fernandes MD et al. [20], who demonstrated a positive correlation between weight gain during pregnancy and serum leptin concentration in all trimesters of pregnancy. The higher leptin concentrations produced during pregnancy are related to weight gain as well as changes in hormone levels, which can stimulate leptin secretion [68]. Fernandes MD et al. [20] also observed a slight decrease in serum leptin concentrations in the second trimester, which was attributed to the development of insulin resistance during this period, as observed in other studies [25,68]. Therefore, it is crucial to demonstrate in future studies whether the slight decrease in leptin concentration in the second trimester is associated with normal gestational weight gain.

In addition to the association of leptin with excessive GWG, we found that percent body fat is also associated, regardless of pBMI. The majority of studies did not assess body fat, which is important since adipokines are secreted by adipose tissue. Only Lacroix et al. [11] included percent body fat in their analysis, finding that leptin is associated with GWG, adjusted by pBMI and body fat. All studies show a greater association between leptin and excessive GWG in individuals living with overweight and obesity compared to those with normal weight. However, when analyzing the percent body fat, we find that the association with pBMI is lost, as the amount of adipose tissue is more important than body weight. It is likely that the reason is leptin is produced and released mainly by adipose tissue into the bloodstream. Blood leptin levels reflect the size of adipose tissue and vary according to nutritional status [69]. Thus, leptin can function as a metabolic regulator linking the body’s nutritional status with processes that require a lot of energy. During pregnancy, energy requirements are essential for adequate maternal weight gain to ensure the development of the fetus, placenta, and other maternal tissues [70]. Another important point related to leptin and reproduction is its secretion by the human placenta, further reinforcing its connection with pregnancy [71]. The formation of the placenta during human gestation is crucial for embryonic development and the success of pregnancy, as it facilitates metabolic exchange and the production of steroids, hormones, growth factors, and cytokines, all critical for maintaining pregnancy [72].

The increase in maternal leptin concentrations is also due to its secretion by the placenta. The mechanisms underlying the role of leptin in weight regulation during pregnancy are not yet well established. Leptin is involved in energy balance, but during gestation, adaptations occur in the central regulation of energy balance, including reduced transport into the brain and leptin resistance. However, these adaptations may not be as well established in individuals with obesity [73].

We found that adiponectin was associated with a lower risk of excessive GWG. This finding is consistent with results observed in adult Mexican women [63]. However, adiponectin showed no association with pre-pregnancy BMI or GWG in Brazilian pregnant adolescents [25]. It is important to note that the Brazilian study included only normal-weight adolescents, suggesting that the association may be present when higher body fat is involved. Adiponectin is secreted by adipocytes and participates in multiple functions such as insulin sensitization, stimulation of lipid metabolism, and glucose absorption, exhibiting anti-inflammatory properties and inversely correlating with body weight and fat mass [20,25]. During pregnancy, adiponectin concentration decreases due to an increase in fat mass.

We did not find any association between irisin and GWG. We hypothesized that since irisin is secreted by both adipose and muscle tissue [26] and is associated with gestational diabetes [27], it might also be associated with GWG. We included physical activity in our analysis, as irisin is stimulated by exercise. However, we found no associations, consistent with findings from another study [15]. This lack of association might be attributed to the generally low levels of physical activity among Mexican adolescents.

Regarding hsCRP, no associations were found with GWG. This result aligns with a study that assessed a panel of inflammatory markers in relation to GWG [13], as well as with the study by Logan et al. [12], who did not observe an association between hsCRP concentrations and gestational weight gain. However, Hrolfsdottir, et al. [35] found a positive association of hsCRP with greater GWG. It is important to note that the samples used in their study were stored for 20 years, suggesting potential differences in nutritional, health, and social environments compared to present-day conditions. While pregnancy is an inflammatory state [32], it appears that adipose tissue functions more in metabolic regulation than in inflammation, suggesting that the association of gestational weight gain with serum leptin concentrations is probably not related to the inflammatory response. Nonetheless, further studies are warranted.

This study has some limitations. Firstly, the participants were recruited at various stages of gestation, resulting in a heterogeneous sample. However, considering that maternal leptin concentrations typically peak in the late second and early third trimester [74], we conducted a sensitivity analysis on a subgroup of participants within a specific range of gestational weeks and found no significant differences in associations. Additionally, only 62.5% of the total sample were included in the analysis due to complete biochemical data availability, potentially affecting the results. Furthermore, body fat was assessed using bioimpedance, which can be influenced by hydration status, particularly at a more advanced gestational age when the volume of amniotic fluid is greater. Nonetheless, comparison with a subsample undergoing skin fold measurements revealed no statistical differences.

Despite these limitations, the study has notable strengths. We evaluated GWG according to IOM recommendations and as a percentage of adequacy, accounting for gestational age, thus providing specific total recommended gestational weight gain calculations. Additionally, we assessed pre-pregnancy BMI accordingly for adolescents, using the WHO growth chart for girls and age. Further, we included factors important for adipokine secretion in our analysis, such as body fat, energy intake, and physical activity.

## 5. Conclusions

In pregnant Mexican adolescents, both leptin and body fat percentage were associated with excessive GWG, independent of pBMI. This suggests that adipose tissue and its adipokines appear to play a more significant role in GWG than pre-pregnancy body weight. Future research should explore the interactions and physiological mechanisms of various adipokines beyond leptin, as well as other inflammatory markers, taking into account body composition and lifestyle factors.

It is essential to promote lifestyles that help adolescents start their pregnancy at an adequate and healthy weight, while providing necessary counseling and support to promote appropriate gestational weight gain.

According to recommendations from the Institute of Medicine of the United States, future research should focus on mechanisms underlying the effects of gestational weight gain on the mother–baby dyad, which may have adverse metabolic consequences later in their lives.

## Figures and Tables

**Table 1:** General characteristics of pregnant adolescents at first visit (*n* = 198).

Characteristics	Variable	*n* (%)
Sociodemographic	Age, years *	15.9 ± 1.4
	Age, group	
	≤15 years	64 (32.3)
	≥16 years	134 (67.7)
	Occupation	
	Homemaker	165 (83.3)
	Student	29 (14.6)
	Employee	4 (2.0)
	Marital status	
	Single	147 (74.2)
	With partner	51 (25.8)
	Socioeconomic level	
	Low	96 (48.5)
	Middle	88 (44.4)
	High	14 (7.1)
	Education, years **	9.0 (8.0–10.0)
Clinical	Menarche, years **	11.6 ± 1.4
	Trimester	
	First	12 (6.1)
	Second	106 (53.5)
	Third	80 (40.4)
Anthropometrical	Pre-pregnancy weight, kg **	52.0 (46.0–57.25)
	Height, cm **	155.0 (151.0–158.3)
	Pre-pregnancy BMI, kg/m^2^ **	21.6 (15.0–33.9)
	Pre-pregnancy BMI	
	Underweight	2 (1.0)
	Normal weight	151 (76.3)
	Overweight	32 (16.2)
	Obesity	13 (6.6)
	GWG, kg **	11.5 (8.8–14.5)
	GWG adequacy, % **	99.1 (78.5–140.6)
	GWG category	
	Inadequate	77 (38.9)
	Adequate	56 (28.3)
	Excessive	65 (32.8)
	Body fat, % *	35.7 ± 5.9
Biochemical	Leptin (ng/mL) **	35.3 (20.6–49.5)
	Adiponectin (ng/mL) **	8627.9 (6065.8–13,275.1)
	Irisin (ng/mL) **	669.1 (380.6–1043.8)
	C-reactive protein (mg/L) **	3.4 (2.0–5.3)
Lifestyle	Physical activity	
	Yes	54 (27.3)
	Energy intake, kcal/d **	1944 (1564–2315)
	Protein adequacy, % **	15.8 (13.0–19.1)
	Lipid adequacy, % **	33.9 (28.0–39.3)
	Carbohydrate adequacy, % **	51.0 (45.0–57.4)

* Mean ± standard deviation, ** Median (percentile 25–percentile 75). BMI: body mass index, GWG: gestational weight gain.

**Table 2:** Cox regression model for inadequate gestational weight gain according to maternal clinical and biochemical characteristics.

	Inadequate Gestational Weight Gain		
Variable	HR	95%CI	*p*-Value
Age (years)	0.87	0.72–1.04	0.129
Leptin (ng/mL)	1.005	0.99–1.01	0.130
Adiponectin (ng/mL)	1.00001	0.99997–1.00005	0.568
Irisin (ng/mL)	1.0004	0.9998–1.001	0.117
hsCRP (mg/L)	1.01	0.98–1.02	0.589
Pre-pregnancy BMI			
Normal weight	Ref.	Ref.	Ref.
Overweight/obesity	0.44	0.18–1.06	0.066
Body fat (%)	0.96	0.91–1.01	0.140
Physical activity			
Yes	Ref.	Ref.	Ref.
No	0.73	0.44–1.19	0.210
Energy intake (kcal/d)	1.0001	0.9998–1.0004	0.415

HR: Hazard Ratio, 95%CI: 95% Confidence Interval, Ref.: category of reference.

**Table 3:** Cox regression model for excessive gestational weight gain according to maternal clinical and biochemical characteristics.

	Excessive Gestational Weight Gain		
Variable	HR	95%CI	*p*-Value
Age (years)	0.89	0.75–1.07	0.223
Leptin (ng/mL)	1.014	1.008–1.021	<0.001
Adiponectin (ng/mL)	0.99994	0.99988–0.99999	0.038
Irisin (ng/mL)	1.0003	0.9997–1.0009	0.279
hsCRP (mg/L)	0.99	0.93–1.05	0.766
Pre-pregnancy BMI			
Normal weight	Ref.	Ref.	Ref.
Overweight/obesity	0.74	0.39–1.40	0.359
Body fat (%)	1.11	1.05–1.17	<0.001
Physical activity			
Yes	Ref.	Ref.	Ref.
No	0.78	0.44–1.31	0.369
Energy intake (kcal/d)	1.0001	0.9997–1.0005	0.408

HR: Hazard Ratio, 95%CI: 95% Confidence Interval, Ref.: category of reference.

## Data Availability

The data presented in this study are available from the corresponding author upon reasonable request. The data are not publicly available due to privacy and ethical reasons.

## Supplementary Materials

The following supporting information can be downloaded at: https://www.mdpi.com/article/10.3390/nu16132147/s1, Table S1: Correlations between anthropometric, biochemical, and dietetic variables in Mexican pregnant adolescents, INPer 2018–2023.
