# Supplementary material for: Total Gestational Weight Gain Is Explained by Leptin and Body Fat, Regardless of Pre-Pregnancy Body Mass Index and Other Adipokines, in Mexican Adolescents
Source: Nutrients. 2024 Jul 5;16(13):2147. doi: 10.3390/nu16132147 (PMC11242962; doi:10.3390/nu16132147)
Supplement: Supplementary file 1 [file nutrients-16-02147-s001.zip › nutrients-3080045-supplementary.pdf]

S1. Correlations between anthropometric, biochemical, and dietetic variables in Mexican pregnant adolescents, INPer 2018-2023.

		Age	BMI z-score	GWG %	Fat mass %	Leptin	Adiponectin	Irisin	CRP	Weeks of gestation*	Energy intake
**Age**	Rho	1.000									
	p-value										
**BMI z-score**	Rho	-0.165	1.000								
	p-value	**0.020**									
**GWG %**	Rho	-0.049	0.242	1.000							
	p-value	0.490	**0.001**								
**Fat mass %**	Rho	-0.104	0.628	0.485	1.000						
	p-value	0.146	**<0.001**	**<0.001**							
**Leptin**	Rho	0.121	0.280	0.257	0.364	1.000					
	p-value	0.090	**<0.001**	**<0.001**	**<0.001**						
**Adiponectin**	Rho	0.126	-0.100	-0.202	-0.207	0.041	1.000				
	p-value	0.077	0.163	**0.004**	**0.003**	0.570					
**Irisin**	Rho	0.153	0.284	0.211	0.328	0.527	-0.090	1.000			
	p-value	**0.031**	**<0.001**	**0.003**	**<0.001**	**<0.001**	0.208				
**CRP**	Rho	0.135	0.263	0.175	0.196	0.181	0.048	0.086	1.000		
	p-value	0.058	**<0.001**	**0.014**	**0.006**	**0.011**	0.506	0.230			
**Weeks of gestation***	Rho	0.041	-0.049	0.007	0.114	0.302	-0.122	0.269	-0.006	1.000	
	p-value	0.570	0.493	0.920	0.111	**<0.001**	0.088	**<0.001**	0.934		
**Energy intake**	Rho	0.084	-0.166	-0.082	-0.156	0.024	0.065	-0.040	0.027	0.104	1.000
	p-value	0.242	**0.020**	0.250	**0.029**	0.733	0.363	0.576	0.709	0.146	

Rho: Spearman’s rho coefficient, BMI: Body mass index, GWG: Gestational weight gain, CRP: C-reactive protein. *Weeks of gestation when the blood sample was obtained.
